# Base-cleavable microarrays for the characterization of DNA and RNA oligonucleotides synthesized *in situ* by photolithography†
†Electronic supplementary information (ESI) available: Experimental procedures for microarray fabrication, deprotection and cleavage as well as LC-MS conditions and spectra of all array eluates. See DOI: 10.1039/c4cc05771f



**DOI:** 10.1039/c4cc05771f

**Published:** 2014-09-12

**Authors:** Jory Lietard, Nicole Kretschy, Matej Sack, Alexander S. Wahba, Mark M. Somoza, Masad J. Damha

**Affiliations:** a Department of Chemistry , McGill University , Montréal , Québec H3A 0B8 , Canada . Email: masad.damha@mcgill.ca; b Institute of Inorganic Chemistry , University of Vienna , 1090 Vienna , Austria . Email: mark.somoza@univie.ac.at

## Abstract

Assessing synthesis efficiency, errors, failed deprotections, and chemical and enzymatic degradation of oligonucleotides on microarrays is essential for improving existing *in situ* synthesis methods, and for the development of new chemistries. We describe the use of LC-MS to analyse DNA and RNA oligonucleotides deprotected and cleaved under basic conditions from microarrays fabricated using light-directed *in situ* chemistry. The data yield essential information on array quality and sequence identity.

## Abstract 2

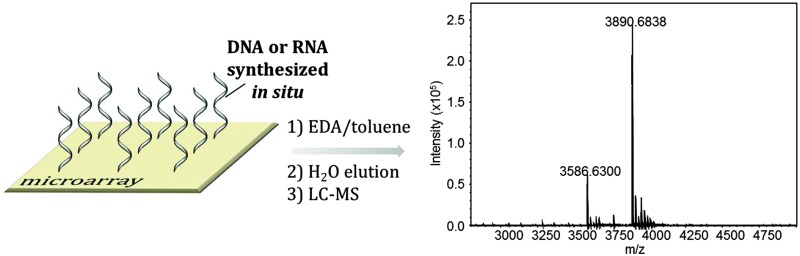
DNA and RNA oligonucleotides synthesized *in situ* on microarrays were cleaved, collected and characterized by LC-MS.

Arraying DNA onto chips has revolutionized the field of biomedical research,^1–4^ most notably in gene expression profiling,^5^ by providing an access to large nucleic acid libraries attached to one single support and by allowing the simultaneous screening of thousands of genes. These DNA libraries can originate from PCR products which are then covalently attached to the glass surface^6^ or are synthesized *in situ* by ink-jet printing or photolithography,^7–9^ taking advantage of the robust phosphoramidite chemistry.^10,11^ The quality of the immobilized DNA is one of the crucial parameters governing the reliability of the measurement,^12^ and while this parameter can be controlled to some extent for PCR products, the same level of quality assessment is less trivial for *in situ*-synthesized microarrays.

One method for quality control consists of labelling the terminus of each strand on the array with a fluorescent nucleotide and measuring the fluorescence intensity.^13,14^ The decrease in intensity as the chain length increases is fitted to an exponential decay curve which then allows for the determination of a stepwise synthesis yield. In addition, this direct labelling and read-out method permits an optimization of the parameters involved in microarray synthesis, thereby enabling a relative control over array quality.^15^ However, fluorescence provides at best a relative measure of sequence completion. The interpretation of the intensity can also be uncertain due to the sequence-dependence of fluorescence,^16^ and it certainly cannot identify the source of error.

To be able to chemically separate the grown oligonucleotides from the glass slide and characterize the eluate using conventional analytical methods is an attractive idea, but the decisively small amount of DNA synthesized on-chip (∼0.1–1 pmol mm^–2^)^17^ requires the most sensitive detection techniques. In this context, radiolabelling of cleaved DNA followed by gel electrophoresis offers an overview of synthetic quality and it has been successfully applied to the monitoring of microarray synthesis defects, but like fluorescence provides primary information on the distribution of sequence lengths.^9,17,18^ Mass spectrometry (MS) is another sensitive method which would provide final evidence of oligonucleotide identity but it has, to our knowledge, only been attempted on microarray surfaces suitable as matrices for MALDI-MS analyses.^19–21^

We therefore wished to develop a method that allows for MS characterization of microarrays fabricated on standard glass microscope slides. In addition to the identification of full-length products, MS would likely detect synthetic failures, degraded material and incompletely deprotected sequences; essential information for the development of new *in situ* chemistries. Indeed, we have recently embarked on the synthesis of RNA microarrays by photolithography^22,23^ and the identification by MS of the synthetic RNA analytes is expected to help guide the technology to maturity. Our approach involved the incorporation of a base-labile ester functionality at the 3′-end of the oligonucleotide chain.^24^ To do so, we used a custom-made NPPOC-protected dT phosphoramidite with a succinyl group attached to the 3′-OH function (cleavable dT, dT^cleav^, Fig. 1a). Following published protocols,^25^ this amidite was coupled for 1 min on silanized glass slides after the synthesis of a pentamer spacer, and the desired oligonucleotide sequence was then fabricated after NPPOC deprotection of the dT^cleav^ (Fig. 1b). To verify that dT^cleav^ coupled efficiently, we labelled the 5′-end of a dT_10_ chain with a Cy3 dye. In parallel, dT decamers fabricated without dT^cleav^ were also fluorescently-labelled. Based on the difference in fluorescence intensity between cleavable and non-cleavable sequences (Fig. S1a, ESI†), an 85% coupling yield was calculated for dT^cleav^. Next, the same arrays were treated in concentrated ammonia at r.t. for 2 h and then scanned. The features where cleavable sequences were synthesized underwent a large drop in fluorescence intensity (Fig. S1b, ESI†), indicating that the ester function was correctly cleaved and release of the oligonucleotide in solution was almost complete.

**Fig. 1:**
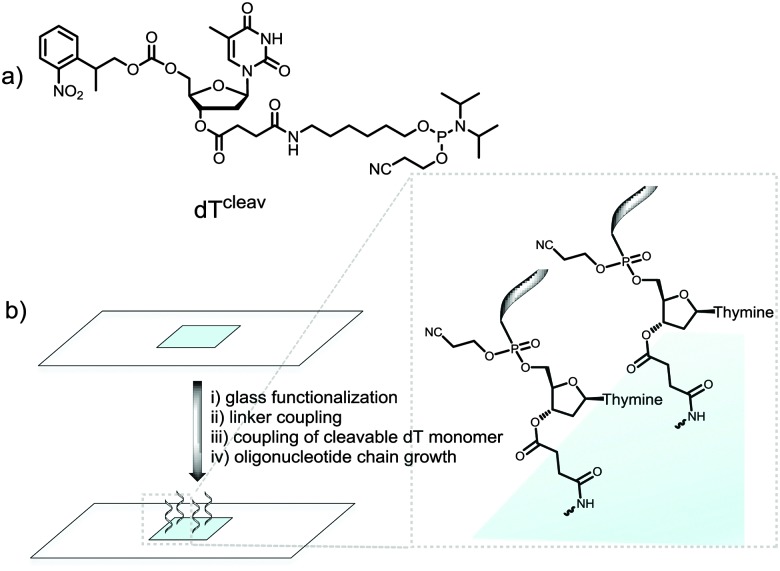
(a) Chemical structure of the cleavable dT monomer; (b) schematic illustration of the synthetic steps involved in the fabrication of microarrays containing a cleavable dT unit. Glass functionalization is performed with a silanizing reagent. The linker is typically a dT or dC pentamer chain.

We then attempted to collect the chemically-cleaved oligonucleotide. We chose to fabricate a simple dT_13_ model sequence according to the procedure depicted in Fig. 1a. After synthesis, the microarray was deprotected in a 1 : 1 mixture of ethylenediamine (EDA) and toluene (Fig. 2a), an alternative to the conventionally employed EDA/ethanol in DNA array deprotection.^8,26^ After 2 h at r.t., the array was thoroughly washed with ACN, dried and the resulting DNA was collected from the surface by applying 100 μl of water (Fig. 2b).

**Fig. 2:**
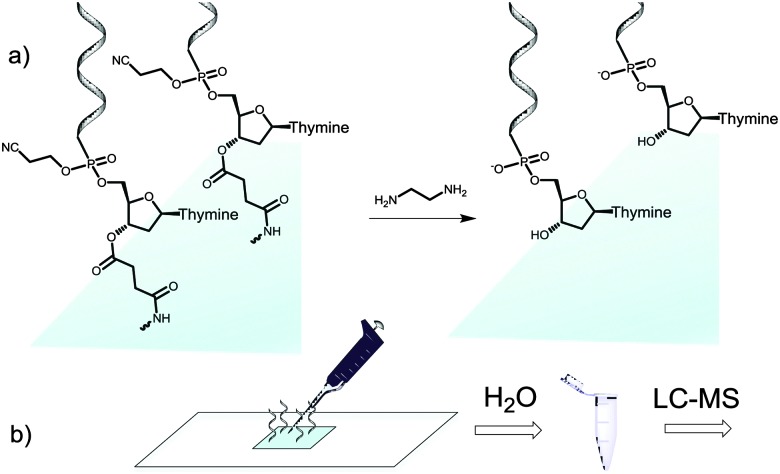
Schematic illustration of the cleave-and-collect process of oligonucleotides synthesized on microarrays. (a) DNA oligonucleotides are first deprotected in EDA/toluene 1 : 1, 2 h, r.t. and the microarray is then washed with ACN (2 × 25 ml); (b) the DNA is then collected by pipetting 100 μl H_2_O over the synthesis area. The microarray eluate is concentrated and analysed by LC-MS.

Quantification of the isolated chip eluate revealed that 20 pmol of material were obtained, consistent with the reported density of available hydroxyl groups on the silanized surface of the substrate.^17^ Using a duplicating method developed earlier in our laboratory where two identical arrays are simultaneously fabricated,^27^ a single automated run yielded up to 40 pmol of deprotected DNA which were subsequently analysed by liquid chromatography (LC)-electrospray ionization (ESI)-MS.

The MS trace of the cleaved dT_13_ is shown in Fig. 3a. The full-length product is detected as a 3′-OH species, demonstrating the correct cleavage at the 3′-ester functionality, together with a significant amount of a shortmer identified as dT_12_. Since the capping step in the synthetic cycle was omitted, the *n* – 1 oligonucleotides are the result of a single failed coupling. In the absence of capping, the oligonucleotide lengths follow a binomial distribution, which allows estimating the coupling yield based on the relative heights of the MS peaks. The relative peak height in Fig. 3a indicates a 98.3% coupling yield for NPPOC-dT; somewhat lower than values previously calculated by the fluorescence method.

**Fig. 3:**
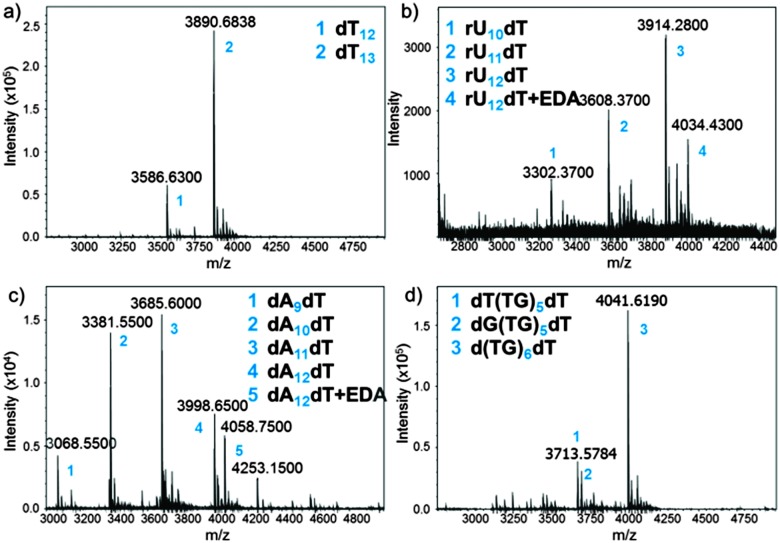
MS spectra obtained after deprotection and cleave-and-collect for the following oligonucleotides: (a) dT_13_; (b) rU_12_dT; (c) dA_12_dT; (d) d(TG)_6_dT. Exact masses are shown. EDA: ethylenediamine. Numbers (blue) are referred to in the inset of each MS spectrum.

Our cleavage method was then applied to the detection of poly dC (Fig. S11, ESI†) and poly dA (Fig. 3c) sequences. Interestingly, the amount of *n* – 1, *n* – 2 and *n* – 3 species in crude poly dA samples exceeds those in poly dT and dC arrays. The full-length product, dA_12_dT, is also present in the form of a noncovalent complex with EDA. Nucleobase deprotection is complete in both dA_12_dT and dC_12_dT cases since no trace of remaining phenoxyacetyl (Pac) or isobutyryl (iBu) groups was detected by MS. The characterization of oligonucleotide arrays was also applied to mixmers of two bases and, as shown in Fig. 3d and Fig. S13 (ESI†), MS resolution allows for the distinction between two different failure sequences.

Inspired by these results and by a previously reported procedure for the complete deprotection of RNA in EDA without facing degradation,^28^ we wished to apply our method to RNA microarrays. A model rU_12_dT array was fabricated using NPPOC 2′-*O*-ALE rU amidites^22^ and was then deprotected as follows: first, decyanoethylation was conducted in Et_3_N/ACN 2 : 3 for 6 h at r.t. then ALE removal was performed in buffered hydrazine hydrate in pyridine/AcOH for 2 h at r.t. The intact succinyl ester was finally cleaved by treating the array with dry EDA/toluene for 2 h at r.t. The crude RNA was eluted from the surface by pipetting a small volume of sterilized water, concentrated, quantified (20 pmol per array) and injected on LC-MS. The MS spectrum is shown in Fig. 3b and the major peak corresponds to the full-length, 3′-OH rU_12_dT, which is flanked by a minor peak at +60 Da resulting from a salt complex with EDA. This measurement offers, for the first time, a direct and concrete proof of correct *in situ* synthesis of RNA microarrays. Compared to dT_13_ in Fig. 3a, larger amounts of *n* – 1 and *n* – 2 species are also detected, which could be due to either failed couplings or to degradation products arising from cleavage at the internucleotidic phosphate. However, the presence of the *n*-mer as the main peak and the lack of 2′,3′-phosphorylated shortmers suggest that degradation is limited.

In an attempt to optimize the quality of *in situ* DNA and RNA microarray fabrication, we envisaged to modify a few key parameters in the design protocols and investigate their effect by MS. We performed this study on the dT_13_ and rU_12_dT models and considered four factors in the synthesis cycle: coupling time, the activator type, capping and oxidation steps. In DNA and RNA microarray synthesis by photolithography, the oxidation of the phosphite triester linkages can be conducted at the latest stage because deblocking the 5′-OH function does not require an acidic solution. The results as well as a representative panel are shown in Fig. 4 and Fig. S4–S10 (ESI†). Including an iodine/water-mediated oxidation or a capping step alone in the synthesis cycle seems to have little effect on array quality (compare Fig. 4b and c to the original array design in Fig. 4a), however when both steps are included, arrays of significantly lower quality were obtained (Fig. 4d). Next, the coupling time was examined and either shortened (from the standard 2 min to 1 min) or extended (5 min). In both DNA and RNA microarrays, shorter or longer coupling times resulted in arrays of poorer quality (Fig. S7, S8, S19 and S20, ESI†). Finally, the conventional 4,5-dicyanoimidazole activator was substituted with tetrazole derivatives, which afforded crude array eluates containing larger amounts of failure sequences (Fig. S9, S10 and S21, ESI†).

**Fig. 4:**
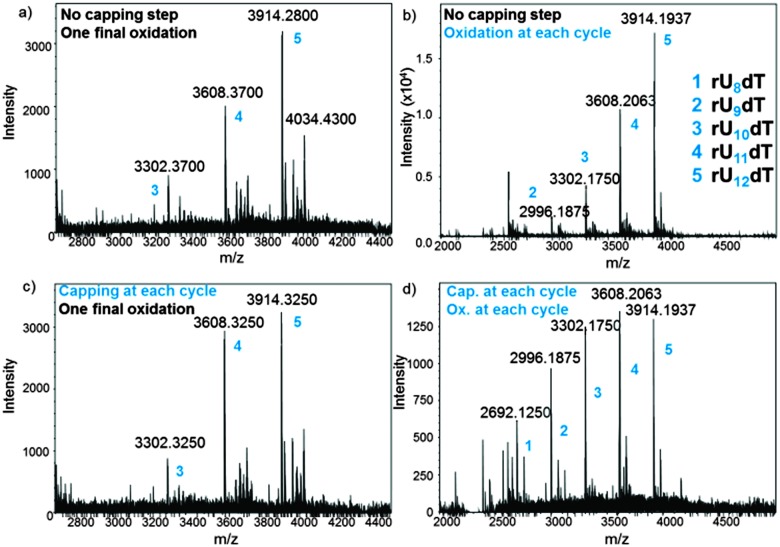
MS spectra obtained after deprotection and cleave-and-collect for rU_12_dT microarrays under various fabrication conditions: (a) standard protocol without capping and oxidation; (b) an oxidation step is included; (c) a capping step is included; (d) both capping and oxidation steps are included. Exact masses are shown. Numbers 1–5 (blue) are all referred to in the MS spectrum (b).

In summary, a reliable protocol for the deprotection and subsequent cleavage of DNA and RNA microarrays with EDA was developed using a 3′-succinylated dT phosphoramidite. The cleaved DNA microarrays or RNA microarrays are insoluble in the deprotection solution and remain on the glass surface,^28^ where they can be collected with water and analysed by LC-ESI-MS. A few picomoles of crude microarray eluates are sufficient to provide a comprehensive overview of chip quality and to monitor the effect of modifying synthesis conditions. Radiolabelling or PCR amplification of the collected DNA/RNA is thus unnecessary. In addition, our approach allows for the first time the assessment of the fidelity of *in situ* RNA microarray synthesis and will have an important impact on the emergence of high-density complex RNA array technology.

The Natural Sciences and Engineering Research Council of Canada (discovery grant to M.J.D.), the Swiss National Science Foundation (Grant #PBBEP2_146174), The Austrian Science Fund (FWF P23797), ChemGenes Corporation and a McGill Fessenden Grant are gratefully acknowledged for financial support. We would also like to thank Dr Jeremy Lackey for fruitful discussions and FlexGen for the generous donation of the cleavable dT monomer.
