# Supplementary material for: Base-cleavable microarrays for the characterization of DNA and RNA oligonucleotides synthesized in situ by photolithography
Source: Chem Commun (Camb). 2014 Sep 12;50(85):12903–6. doi: 10.1039/c4cc05771f (PMC4183992; doi:10.1039/c4cc05771f)
Supplement: Supplementary file 1 [file CC-050-C4CC05771F-s001.pdf]

Electronic Supplementary Material (ESI) for ChemComm.
This journal is © The Royal Society of Chemistry 2014

Base-cleavable microarrays for the characterization of DNA and RNA oligonucleotides synthesized *in situ* by photolithography

Jory Lietard,[a,b] Nicole Kretschy,[b] Matej Sack,[b] Alexander S. Wahba,[a] Mark M. Somoza[b] and Masad J. Damha[a]

Supplementary material

Table of Contents

General experimental section	S2
Microarrays	S2
General fabrication procedures for *in situ* DNA and RNA arrays	S2-S3
Deprotection procedures for DNA and RNA arrays	S4
Coupling of the cleavable dT monomer - Determination of the cleavage efficiency	S4-S5
Microarray cleave-and-collect procedure	S7
Mass spectrometry	S7
LC-MS conditions	S7
MS traces	S8
dT_{13}	S8-S10
$dC_{12}dT$	S10
$dA_{12}dT$, $d(TA)_6dT$, $d(TG)_6dT$	S11
$rU_{12}dT$	S12-S14
Quantification of the isolated material	S14
References	S15

General experimental section

All anhydrous solvents were purchased from Sigma-Aldrich and Biosolve and stored on 4Å molecular sieves. All chemicals and reagents were purchased from either Sigma-Aldrich or ChemGenes and used without further purification. DNA phosphoramidites were purchased from Sigma-Aldrich (SAFC) and RNA phosphoramidites from ChemGenes.

Microarrays

General fabrication procedures for in situ *DNA and RNA arrays*

Microarrays using Maskless Array Synthesis (MAS) were fabricated as described previously.[1-3] Briefly, for paired array synthesis, Schott Nexterion Glass D slides were first drilled with a 0.9 mm diamond bit, washed and rinsed in an ultrasonic bath. The slides were then silanized using *N*-(3-triethoxysilylpropyl)-4-hydroxybutyramide (Gelest SIT8189.5): a metal rack containing 500 ml of a 95:5 (v/v) ethanol/H_2O, 1 ml of acetic acid and 10 mg of the silanizing reagent was loaded with the glass slides and gently agitated for 4h. The slides were then transferred into a similar solution without silanizing reagent, washed by gently agitating for 20 min, removed and then cured in a preheated vacuum oven at 120 °C overnight. After silanization, the slides were stored in a dessicator cabinet until further use.

Microarrays were synthesized directly on the slides using a Maskless Array Synthesizer which consists of an optical imaging system that uses a digital micromirror device to deliver patterned ultraviolet light near 365 nm to the synthesis surface. Under patterned UV irradiation, the selective removal of the photolabile 2-(2-nitrophenyl)-propyloxycarbonyl (NPPOC) 5'-OH protecting group governs the layout and the sequences of the oligonucleotides on the microarray. Reagent delivery and exposure to UV light are synchronized and controlled by a computer coupled to an Expedite 8909 synthesizer (Perspective Biosystems) which performs the actual delivery of solvents and reagents to the array synthesis chamber. The phosphoramidite chemistry employed is similar to that used in standard automated solid-phase synthesis with the exception of the NPPOC group which is removed by UV

irradiation near 365 nm in presence of a solution of 1% (m/v) imidazole in DMSO. Light exposure is performed using a radiant energy density of 6.7 J/cm^2 (typical exposure times of 97 sec. at 80mw/ cm^2).

DNA amidites containing a 5'-NPPOC group and, as base protecting groups, isobuyryl (iBu) for dC, *tert*-butylphenoxyacetyl (Tac) for dA and isopropylphenoxyacetyl (iPac) for dG were used. For RNA amidites, 5'-NPPOC 2'-*O*-acetal levulinyl ester (ALE) monomers were used, with phenoxyacetyl (Pac) for rA and rG and iBu for rC as base protecting group. For the coupling step, amidites were prepared as 0.03 M solutions in dry ACN for DNA and 0.05 M in dry ACN for RNA and the coupling step was set to 2 min by default.

Cleavable microarrays were fabricated according to the following steps:

1) Functionalization of the silanized substrate with a dT_5 or a dC_5 spacer
2) Coupling of the cleavable dT monomer
3) Coupling of the next 12 phosphoramidites (DNA or RNA)

Microarrays were scanned using a GenePix 4400A (Molecular Devices) and data was extracted with NimbleScan (Roche-NimbleGen) and Excel.

Deprotection procedures for DNA and RNA arrays

Non-cleavable DNA microarrays were deprotected at r.t. for 2 h in a 1:1 mixture of EDA/EtOH (50 ml in a staining glass jar), washed with ACN (2 x 40 ml), dried by blowing a stream of argon then scanned or stored in a dessicator cabinet until further use. RNA microarrays (cleavable and non-cleavable) were first deprotected in Et_3N/ACN 2:3 for 6 h at r.t. (40 ml in a falcon tube) then rinsed in ACN (2 x 40 ml) and dried with argon. The array was then gently agitated for 2 h at r.t. in a 0.5 M hydrazine hydrate solution in pyridine/AcOH 3:2 (40 ml in a falcon tube) to effect the removal of the 2'-ALE group. The array was then washed with ACN (2 x 40 ml) and dried with Argon before proceeding with microarray cleavage.

Coupling of the cleavable dT monomer - Determination of the cleavage efficiency

The cleavable dT monomer was prepared as a 0.03 M solution in dry ACN and coupled for 2 min. After coupling, the capping and oxidation steps were not included. To determine the coupling efficiency as well as verifying the cleavage efficiency of dT^{cleav} under basic conditions, a specific microarray layout was designed. After coupling of a dT_5 spacer on the slide, half of the oligonucleotides received a dT^{cleav} unit and the other half a standard dT monomer. All oligonucleotides then were coupled with regular dT amidites to obtain dT_{11} or $dT^{cleav}dT_{10}$ sequences. This process was repeated twice only on the sequences having coupled with a dT^{cleav} unit, so as to synthesize the following sequences on a single microarray (dT_5 spacers are omitted for clarity; sequences are shown 3' to 5'):

- dT_{11}
- $dT^{cleav}dT_{10}$
- dT_{22}
- $dT^{cleav}dT_{10}dT^{cleav}dT_{10}$
- dT_{33}
- $dT^{cleav}dT_{10}dT^{cleav}dT_{10}dT^{cleav}dT_{10}$

Half of those sequences then received a final coupling with a fluorescent dye (Cy3) while the other half remained as 5'-OH species and will be used for background substraction. After Cy3 coupling, the array was washed in ACN for

2h, dried then scanned. The array was then deprotected and cleaved with concentrated ammonia (1 ml in a polyethylene microarray storage unit) for 2 h at r.t. under gentle agitation. The ammonia solution was removed and the array washed with water then dried by centrifugation and scanned. Scanned arrays before and after ammonia-mediated cleavage are shown in Figure S1. A uniformly bright fluorescent pattern was obtained after synthesis (Figure S1a) and, after treatment of the cleavable array in ammonia, a large amount of bright features gave way to dark spots (Figure S1b) indicating cleavage happening solely on oligonucleotides containing one or multiple dT^{cleav} units. After data extraction, the fluorescence intensities of all microarray features were plotted in Figure S2.

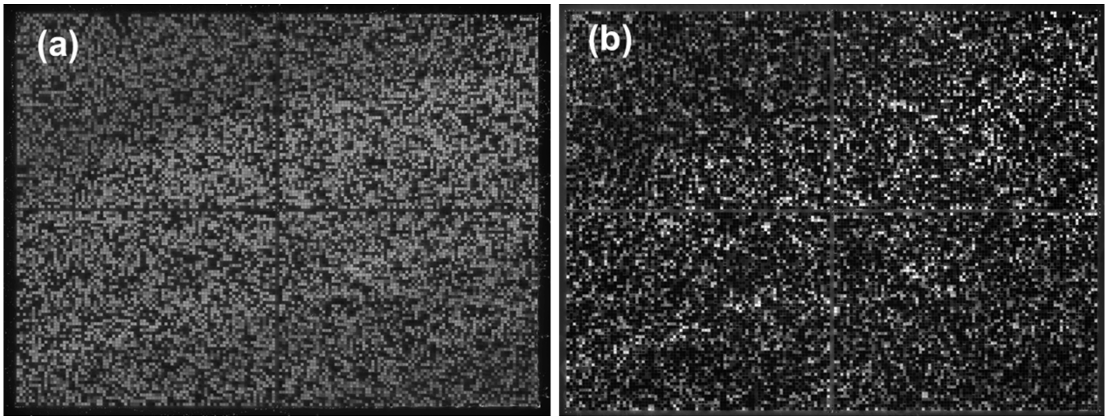

Figure S1. Scanned cleavable microarrays on a fluorescence scanner before (a) and after (b) ammonia-mediated cleavage.

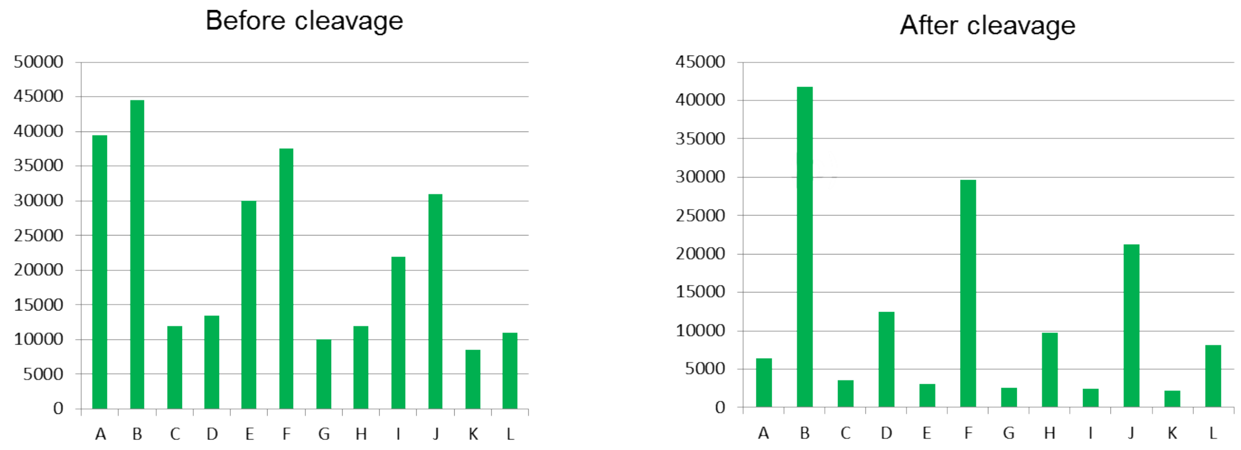

A = $dT^{cleav}dT_{10}$**Cy3** E = $dT^{cleav}dT_{10}dT^{cleav}dT_{10}$**Cy3** I = $dT^{cleav}dT_{10}dT^{cleav}dT_{10}dT^{cleav}dT_{10}$**Cy3**

B = dT_{11}**Cy3** F = dT_{22}**Cy3** J = dT_{33}**Cy3**

C = $dT^{cleav}dT_{10}$ G = $dT^{cleav}dT_{10}dT^{cleav}dT_{10}$ K = $dT^{cleav}dT_{10}dT^{cleav}dT_{10}dT^{cleav}dT_{10}$

D = dT_{11} H = dT_{22} L = dT_{33}

Figure S2. Plotted fluorescence intensities of sequences grown on a cleavable microarray before and after cleavage by ammonia.

Before scanning, the shortest cleavable sequences showed good fluorescent intensities, only slight lower than those of the same lengths without dT^{cleav}. The fluorescence intensity decreased as the chain length increased, but the difference in intensity between cleavable and non-cleavable sequences remained comparable. Using the equation Eq. 1 (where A, B, C and D represent the fluorescence intensities of the corresponding sequences), the coupling efficiency was estimated at 85% for dT^{cleav}.

$$\rho = \frac{A - C}{B - D} \qquad \textbf{Eq. 1}$$

Microarray cleave-and-collect procedure

Cleavable DNA and RNA microarrays were cleaved by treating both arrays out of a single MAS run in 1:1 EDA/toluene for 2 h at r.t. The arrays were then washed with ACN (2 x 25 ml) and dried by blowing a stream of Argon. Sterilized MilliQ-H_2O (MQ-H_2O; 100 µl) was then applied on top of each of the now visible synthesis area and pipetted back and forth to ensure complete retrieval of the cleaved DNA or RNA. The chip eluate was then collected in an eppendorf, evaporated to dryness and rediluted in 10 µl MQ-H_2O. The cleaved material was then quantified using a NanoDrop Lite spectrophotometer (ThermoScientific).

To eliminate ethylenediammonium adducts observed by MS, the microarray eluates were desalted using ZipTip C_{18} pipette tips (Millipore) according to the manufacturer's protocol. Briefly, the tips were wetted by aspiring 2 x 10 µl 1:1 ACN/H_2O then equilibrated by aspiring 2 x 10 µl 0.1 M triethylammonium acetate (TEAA) buffer. The eluate, prepared as a 0.1 M TEAA solution in 10 µl MQ-H_2O, was bound to the C_{18} support by aspiring and emptying the tip 10 times. The tip was then washed with 0.1 M TEAA (3 x 10 µl) and H_2O (3 x 10 µl) and the material was eluted by washing the pipette tip with 10 µl 1:1 ACN/H_2O. The recovered, desalted oligonucleotide was evaporated to dryness and redissolved in 10 µl MQ-H_2O.

Mass spectrometry

LC-MS conditions

The isolated microarray eluates were analyzed by LC-MS using a Dionex Ultimate 3000 UHPLC coupled to a Bruker Maxis Impact QTOF mass spectrometer in negative ESI mode. Samples were run through an Acclaim RSLC 120 C_{18} column (2.2 µm 120A 2.1 x 50 mm) using a gradient of 98% mobile phase A (100 mM HFIP and 5 mM TEA in H_2O) and 2 % mobile phase B (MeOH) to 40 % mobile phase A and 60% mobile phase B in 8 minutes. The data was processed and deconvoluted using the Bruker DataAnalysis software version 4.1.

MS traces

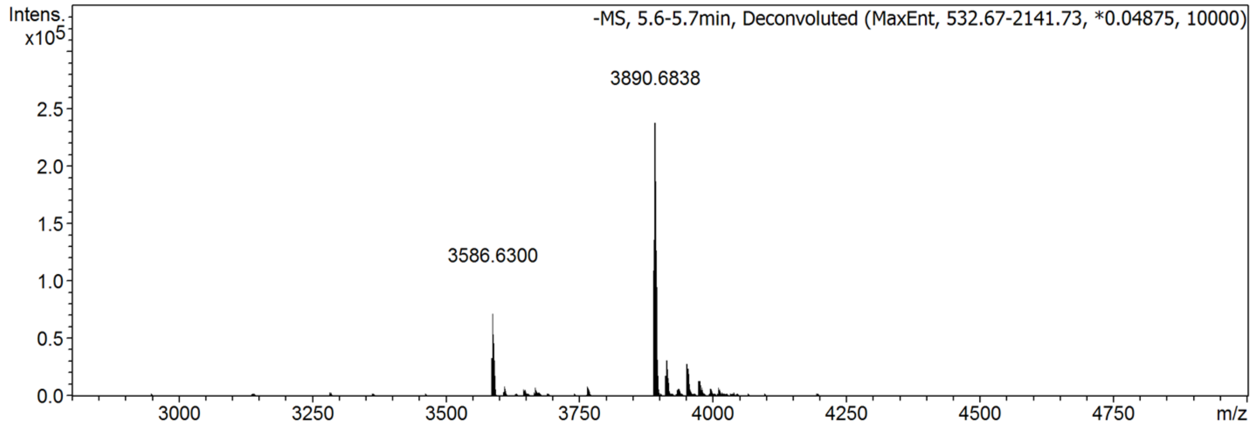

Figure S3. dT_{13} (M_{calcd} = 3890.64 Da) – standard synthesis protocol

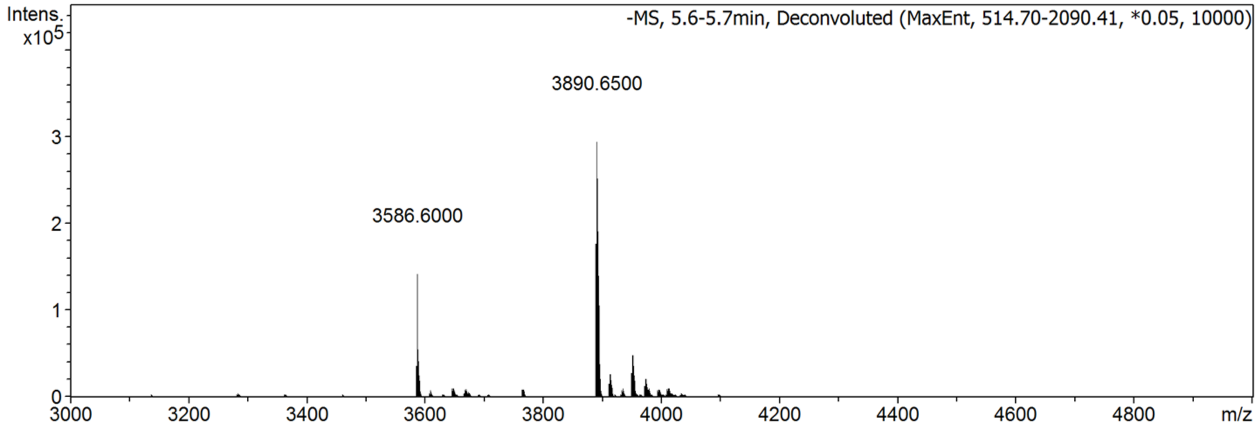

Figure S4. dT_{13} – protocol with a capping step at each synthetic cycle

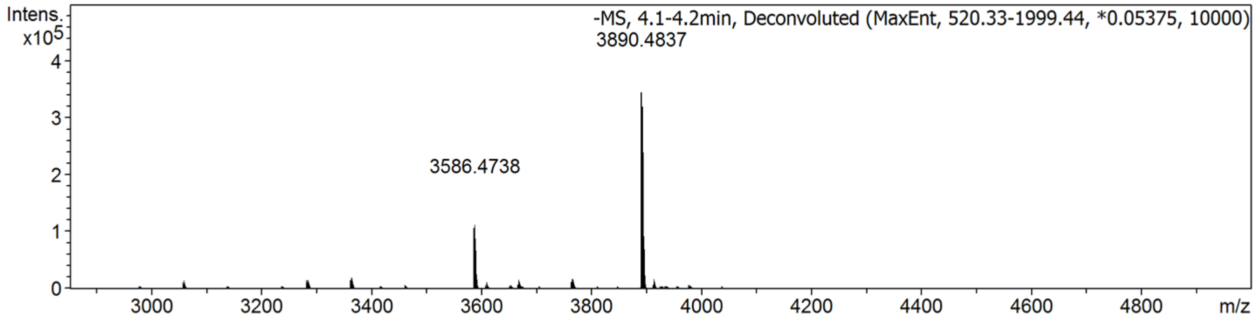

Figure S5. dT_{13} – protocol with an oxidation step at each synthetic cycle

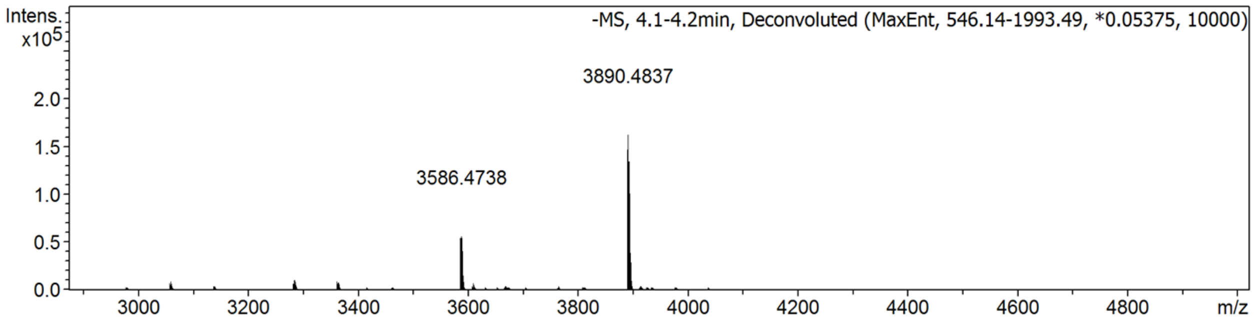

Figure S6. dT_{13} – protocol with oxidation and capping at each synthetic cycle

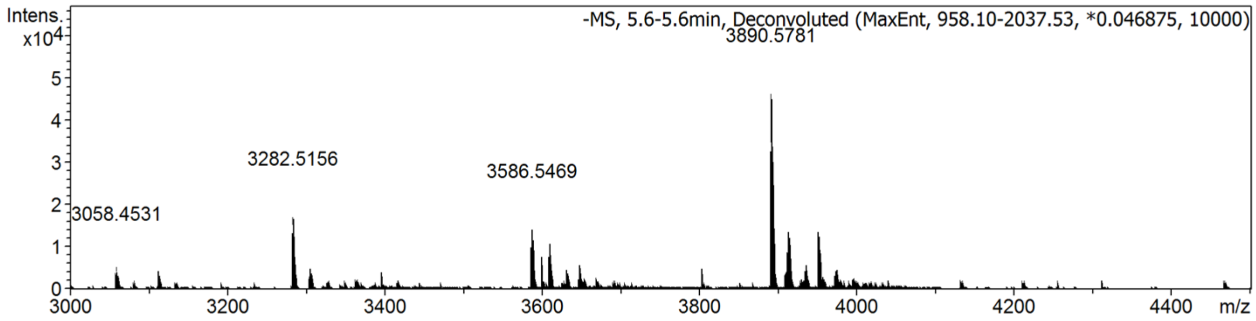

Figure S7. dT_{13} – standard protocol with a coupling time of 5 min for dT. Peak at m/z 3282 corresponds to dT_{11} (M_{calcd} = 3282.55 Da)

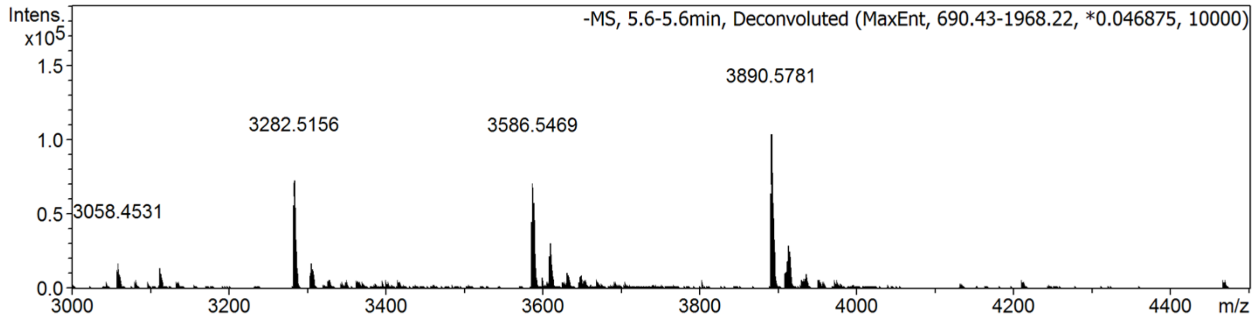

Figure S8. dT_{13} – standard protocol with a coupling time of 1 min for dT

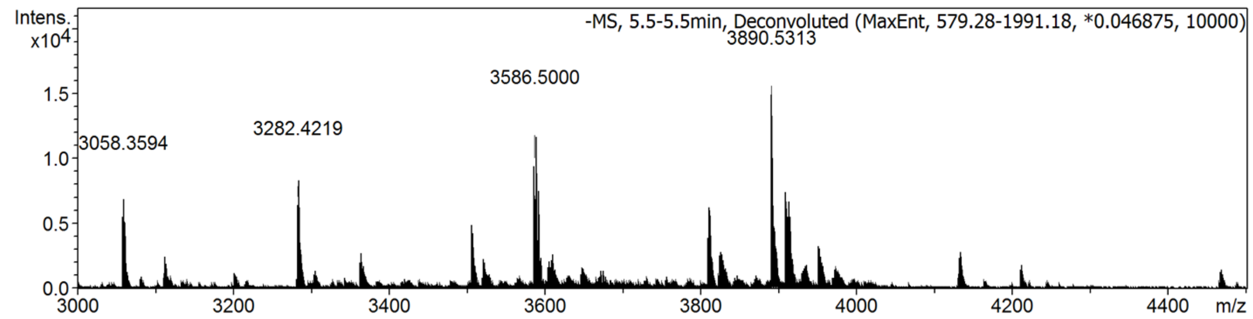

Figure S9. dT_{13} – standard protocol with benzylthiotetrazole (BTT) as activator (instead of DCI)

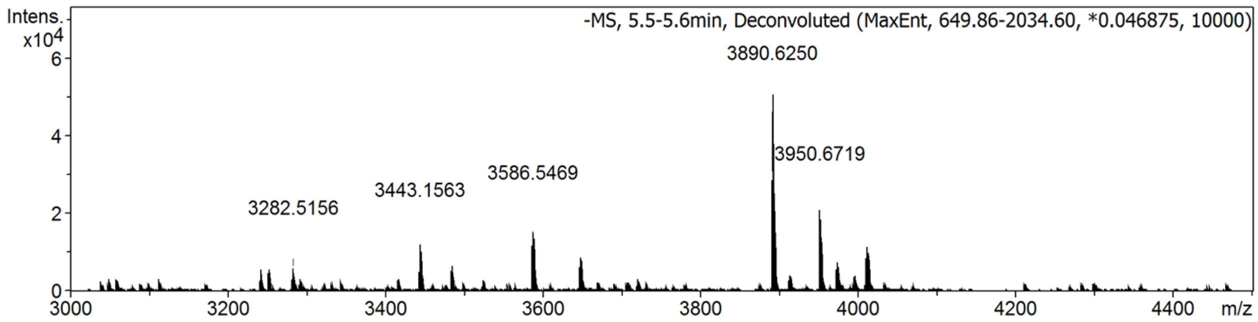

Figure S10. dT_{13} – standard protocol with 5-[3,5-bis(trifluoromethyl)phenyl]-1*H*-tetrazole (Activator 42®) as activator. Peak at m/z 3950 corresponds to an EDA adduct with dT_{13}

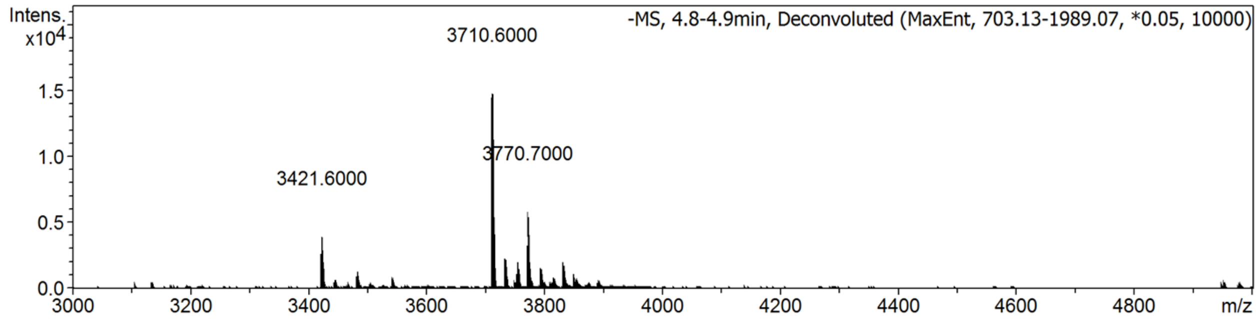

Figure S11. $dC_{12}dT$ (M_{calcd} = 3712.66 Da) – standard synthesis protocol. Peak at m/z 3421 corresponds to $dC_{11}dT$ (M_{calcd} = 3423.62 Da). Peak at m/z 3770 corresponds to an EDA adduct with $dC_{12}dT$

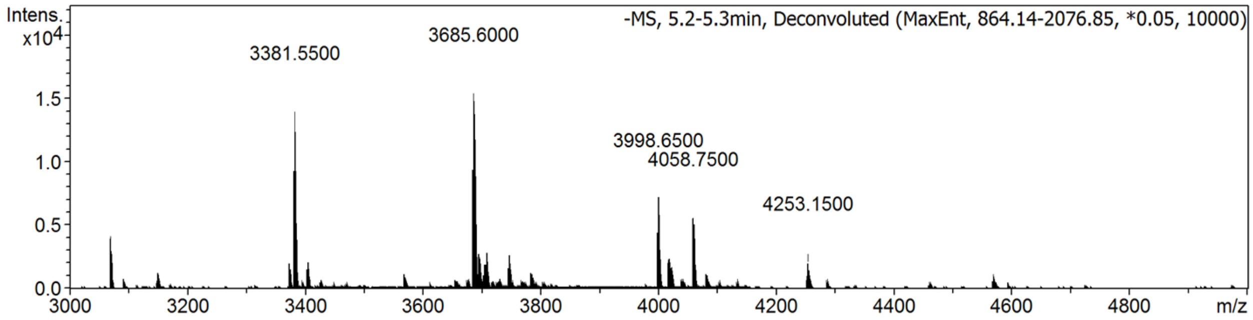

Figure S12. $dA_{12}dT$ (M_{calcd} = 3998.78 Da) – standard synthesis protocol

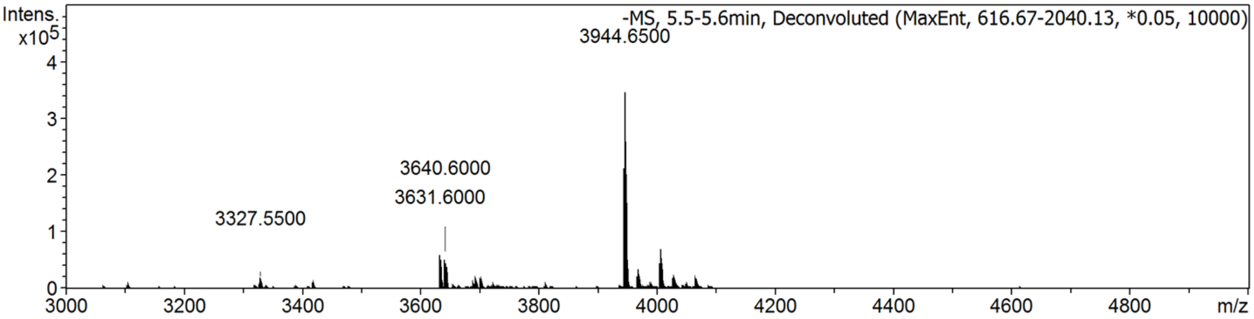

Figure S13. $d(TA)_6dT$ (M_{calcd} = 3944.71 Da) – standard synthesis protocol. Peak at m/z 3327 corresponds to $d(TA)_5dT$ (M_{calcd} = 3327.61 Da). Peak at m/z 3631 corresponds to $dTd(TA)_5dT$ (M_{calcd} = 3631.65 Da). Peak at m/z 3640 corresponds to $dAd(TA)_5dT$ (M_{calcd} = 3640.67 Da)

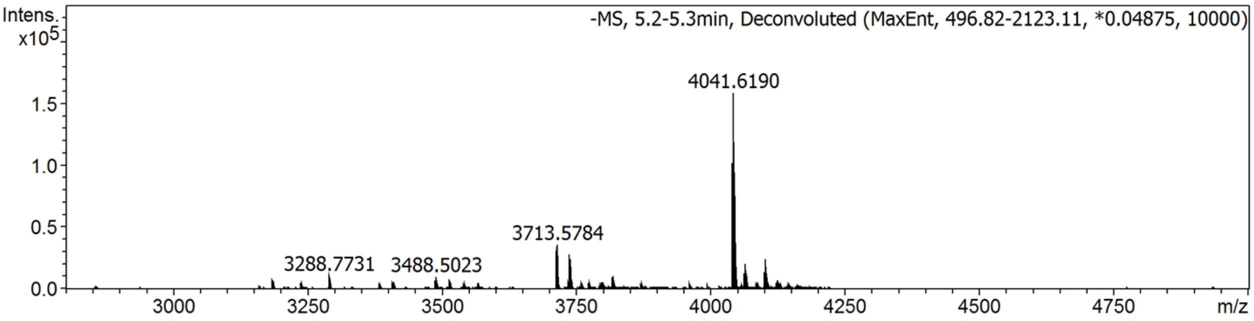

Figure S14. $d(TG)_6dT$ (M_{calcd} = 4040.68 Da) – standard synthesis protocol

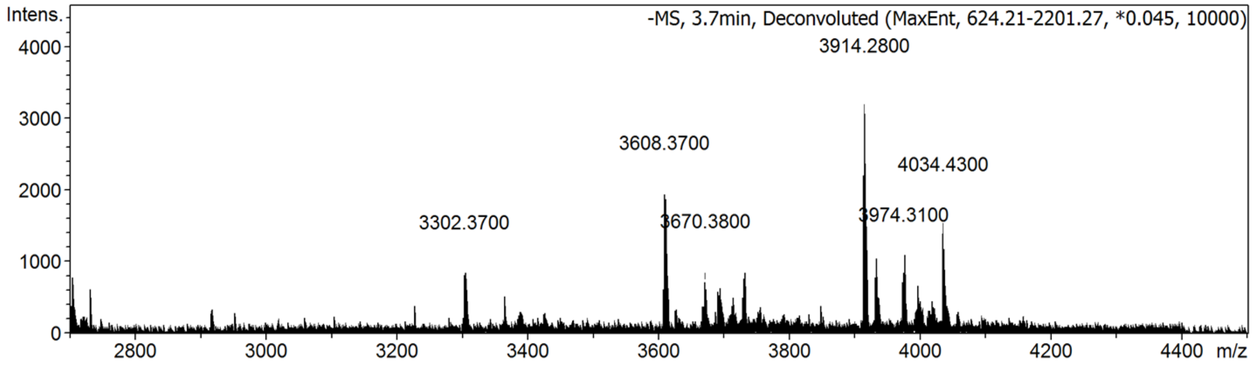

Figure S15. $rU_{12}dT$ (M_{calcd} = 3914.39 Da) – standard synthesis protocol

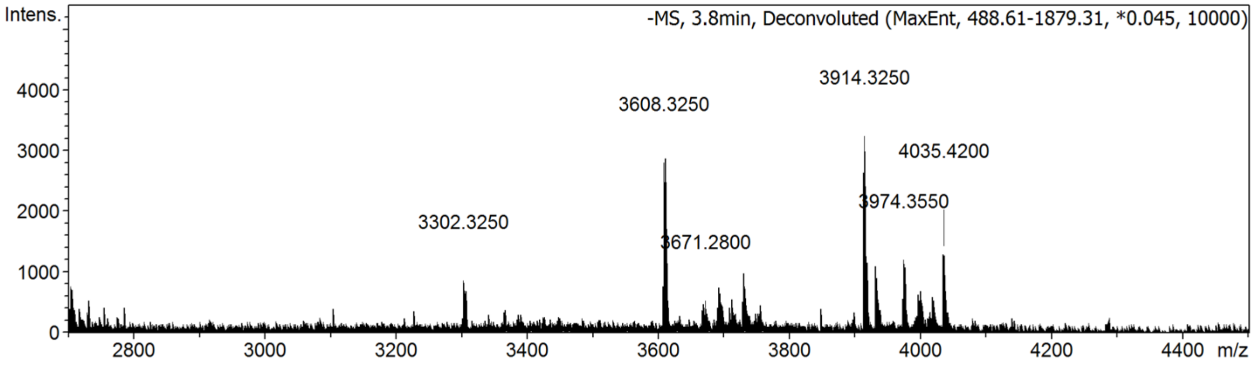

Figure S16. $rU_{12}dT$ – protocol with a capping step at each synthetic cycle. Peak at m/z 3974 corresponds to a single EDA adduct with $rU_{12}dT$

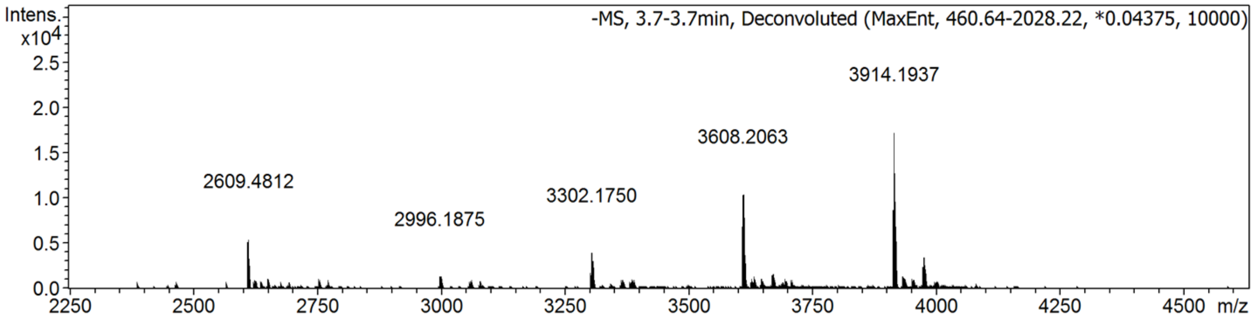

Figure S17. $rU_{12}dT$ – protocol with an oxidation step at each synthetic cycle. Peak at m/z 2609 is an artefact of deconvolution.

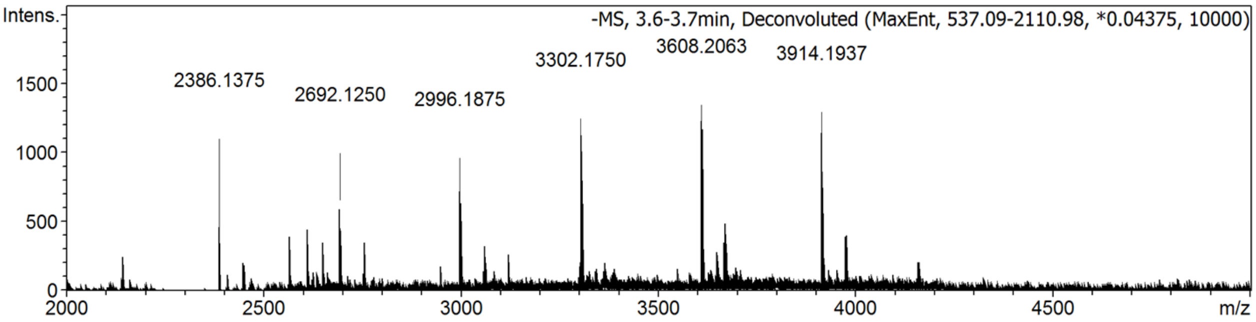

Figure S18. rU$_{12}$dT – protocol with oxidation and capping at each synthetic cycle. Peak at m/z 2386 corresponds to rU$_7$dT (M_{calcd} = 2384.27 Da)

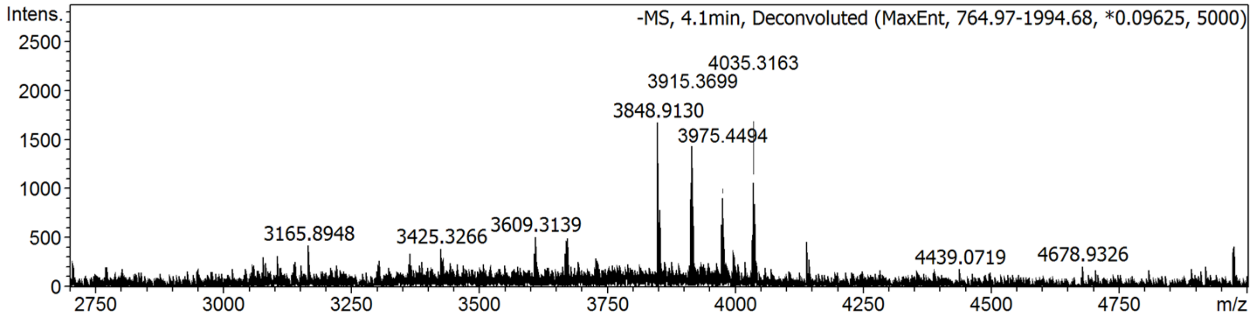

Figure S19. rU$_{12}$dT – standard protocol with a coupling time of 5 min for rU. Peak et m/z 3848 is an artefact of deconvolution. Peaks at m/z 3975 and 4035 are EDA adducts with rU$_{12}$dT

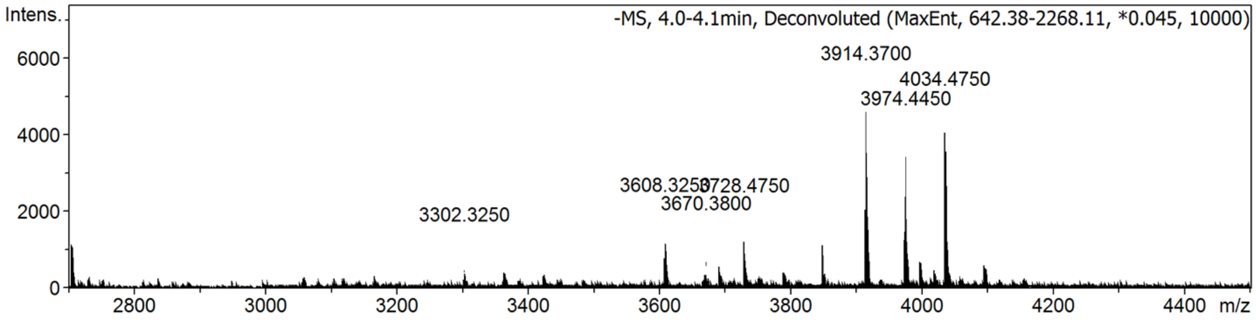

Figure S20. rU$_{12}$dT – standard protocol with a coupling time of 1 min for rU

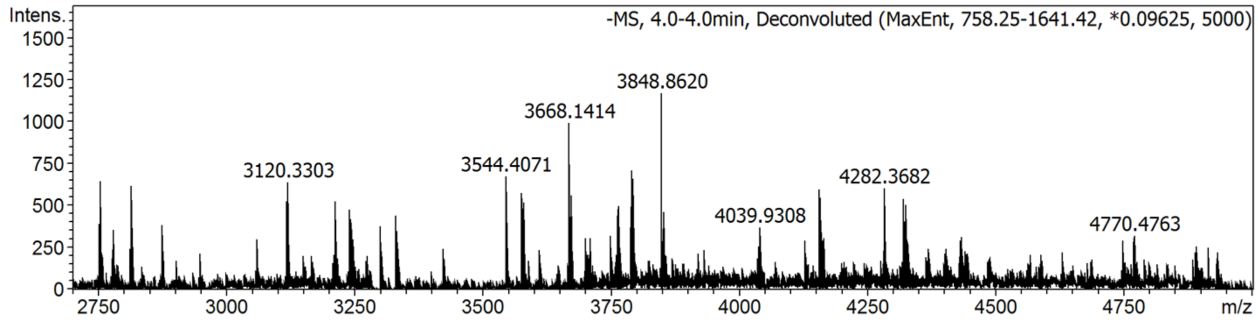

Figure S21. $rU_{12}dT$ – standard protocol with Activator 42® as activator

Quantification of the isolated material

Cleaved oligonucleotides	*Isolated quantity (per array)*	Cleaved oligonucleotides	*Isolated quantity (per array)*	Cleaved oligonucleotides	*Isolated quantity (per array)*
**dT_{13}**		**$dC_{12}dT$**	6 pmol	**$rU_{12}dT$**	
Standard protocol	21 pmol	**$dA_{12}dT$**	12 pmol	Standard protocol	19 pmol
With capping step	56 pmol	**$d(TA)_6dT$**	21 pmol	With capping step	16 pmol
With oxidation step	59 pmol	**$d(TG)_6dT$**	27 pmol	With oxidation step	19 pmol
With capping and oxidation	27 pmol			With capping and oxidation	21 pmol
1 min dT coupling time	20 pmol			1 min rU coupling time	22 pmol
5 min dT coupling time	8 pmol			5 min rU coupling time	4 pmol
With BTT as activator	16 pmol			With Activator 42® as activator	7 pmol
With Activator 42® as activator	22 pmol				

References

1. J. G. Lackey, D. Mitra, M. M. Somoza, F. Cerrina and M. J. Damha, J. Am. Chem. Soc., 2009, **131**, 8496-8502.
2. C. Agbavwe, C. Kim, D. Hong, K. Heinrich, T. Wang and M. M. Somoza, J. Nanobiotechnol., 2011, **9**.
3. M. Sack, N. Kretschy, B. Rohm, V. Somoza and M. M. Somoza, Anal. Chem., 2013, **85**, 8513-8517.
